# Small-angle neutron scattering from CuCrZr coupons and components

**DOI:** 10.1107/S1600576721008311

**Published:** 2021-09-13

**Authors:** F. Schoofs, S. King, A. J. Cackett, M. Leyland, C. Hardie

**Affiliations:** a United Kingdom Atomic Energy Authority, Culham Science Centre, Abingdon OX14 3DB, United Kingdom; b ISIS Pulsed Neutron and Muon Source, Rutherford Appleton Laboratory, Harwell Campus, Didcot OX11 0QX, United Kingdom

**Keywords:** small-angle neutron scattering, SANS, alloys, CuCrZr

## Abstract

Small-angle neutron scattering (SANS) is performed to analyse the microstructural state of a reference CuCrZr material with carefully controlled heat treatments, small-scale manufacturing mock-ups of assemblies and high-heat-flux-exposed mock-ups for fusion reactor components. The information derived from the SANS data corresponds well to existing literature data based on microscopic-scale techniques, but is obtained at millimetre scale with minimal surface preparation. The manufacturing method and high-heat-flux testing conditions are confirmed to have little impact on the microstructural properties, demonstrating the validity of these treatments for scaled-up reactor components.

## Abstract 2

Small-angle neutron scattering (SANS) is performed to analyse the microstructural state of a reference CuCrZr material with carefully controlled heat treatments, small-scale manufacturing mock-ups of assemblies and high-heat-flux-exposed mock-ups. The work demonstrates that SANS can be used for millimetre-scale analysis of small engineering components with minimal surface preparation.

## 1. Introduction   

CuCrZr alloys are favoured in applications where a high thermal conductivity and high strength are required at elevated operating conditions, such as in heat exchange structures in nuclear fusion reactors, for example ITER and EU-DEMO (Kalinin & Matera, 1998 ▸; Kalinin *et al.*, 2007 ▸; Coenen *et al.*, 2016 ▸). Precipitation hardening, with precipitate dimensions in the 1–100 nm range, is crucial to their mechanical strength during operation and therefore needs to be extremely well controlled (Ivanov *et al.*, 2002 ▸; Cackett *et al.*, 2018 ▸). During manufacturing and use, these components are exposed to complex non-uniform thermal cycles, which will impact on the CuCrZr microstructure. Atom probe tomography (APT) or transmission electron microscopy (TEM) can provide direct visual information, but only on a very small sample (<10 µm^3^) (Chbihi *et al.*, 2012 ▸; Hatakeyama *et al.*, 2008 ▸; Jha *et al.*, 2021 ▸; Chen, Jiang, Jiang *et al.*, 2018 ▸; Edwards *et al.*, 2007 ▸). Small-angle neutron scattering (SANS), on the other hand, is able to provide a bulk statistical measurement on millimetre-scale samples, while maintaining the sensitivity for the precipitate dimensions (Schmidt, 1991 ▸; Vogel, 2013 ▸). Here, we compare SANS data for different ageing conditions of a CuCrZr alloy in small (centimetre-scale) samples as well as in bulk components (∼10 cm) exposed to different temperature cycles as part of the EU-DEMO ‘thermal break’ divertor exhaust mock-up development process (Barrett *et al.*, 2015 ▸; You *et al.*, 2018 ▸; Fursdon *et al.*, 2017 ▸; Lukenskas *et al.*, 2019 ▸).

## 2. Experimental   

### 2.1. Materials   

In this work we discuss three different sample sets: reference coupons of CuCrZr with controlled heat treatment; CuCrZr pipes with a single block of W brazed to them, used as manufacturing mock-ups (MUPs); and CuCrZr pipes with multiple W blocks brazed to them, which have been exposed to high heat fluxes. In all cases the starting material was a solution-annealed CuCrZr alloy (CuCrZr 2.1293; Zollern GmbH & Co. KG), with a composition of 1 wt% Cr, 0.06 wt% Zr and a P content of less than 0.005 wt%. The average grain size in the as-received state is 77.9 µm, according to the material’s certificate. Through careful annealing, it is possible to achieve a microstructure containing finely dispersed Cr-rich β′-phase precipitates, which result in peak performance with respect to mechanical properties (Chen, Jiang, Jiang *et al.*, 2018 ▸).

#### 2.1.1. Reference coupons   

Sections of the starting material were heat treated in vacuum for 2 h at 673, 753, 823, 873, 923 and 973 K. The 753 K condition corresponds to the optimal (peak) ageing time and temperature for this alloy with respect to its mechanical properties (Merola *et al.*, 2002 ▸; Kalinin *et al.*, 2007 ▸). The conditions at the lower or higher temperatures represent, respectively, under- or over-ageing which results, in either case, in sub-optimal mechanical performance. Material coupons of 15 × 15 × 1 mm were cut for all heat treatments, as well as 0.5 and 1.5 mm-thick samples of the as-received and 973 K treatment materials. The data on the latter samples are not shown here, but no thickness effects on the SANS data were observed.

#### 2.1.2. Manufacturing mock-ups   

**Figure 1:**
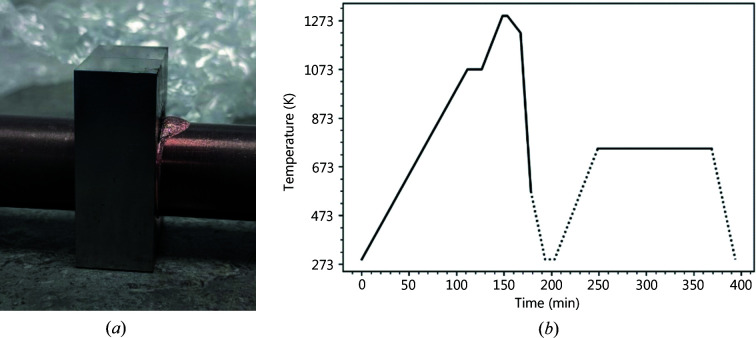
(*a*) Photograph of MUP B after brazing and before sample preparation, showing the CuCrZr tube passing through a W monoblock. (*b*) Furnace cycle for the MUPs.

For each MUP sample, a single rolled tungsten block, with dimensions 12 × 23 × 30 mm and a central bore of 19 mm, containing a 1 mm cast Cu interlayer (ALMT, Japan) was vacuum brazed using an Au–Cu braze to a 15 mm-diameter, 1.5 mm-thick CuCrZr pipe as previously described (Lukenskas *et al.*, 2019 ▸). The brazing cycle for the MUPs is shown in Fig. 1 ▸(*b*) – the difference between MUPs A and B is that the CuCrZr pipe in ‘B’ has undergone this cycle twice, to improve braze flow and adhesion after removal of the segregated Zr at the surface according to prior experience at the UK Atomic Energy Authority with brazing this alloy (Fursdon *et al.*, 2017 ▸; Lukenskas *et al.*, 2019 ▸).

**Figure 2:**
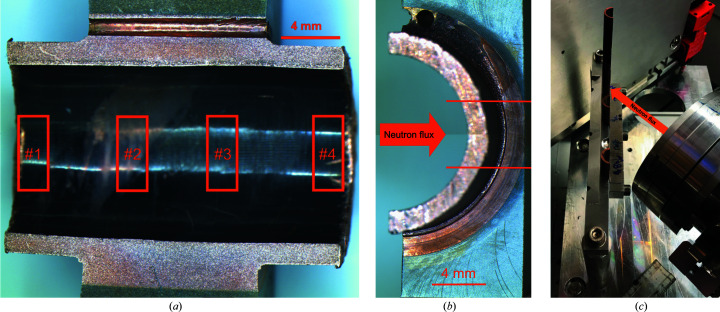
(*a*) Cross section of MUP B, with the SANS (2 × 5 mm rectangles) measurement positions indicated. (*b*) Side view of MUP B, showing the SANS measurement positions with the 5 mm high beam. (*c*) View of the pipe sections installed on the ZOOM instrument. The steel safety shutter behind the samples is raised during the measurements. The samples could be rotated and translated in the neutron beam. In the image the right-hand edge of the right-hand pipe section has been rotated into normal incidence to the incoming neutron beam.

The MUPs were cut lengthwise by wire electrical discharge machining to expose the pipe cross section. An additional cut was made to remove the tungsten and expose the Cu interlayer. The remaining Cu thickness was measured to account for it in the SANS data reduction. No further sample preparation was done on the SANS samples. On each MUP, four measurement locations were investigated: two on the pipe away from and two underneath the tungsten monoblock (see Fig. 2 ▸). SANS measurements were taken with a 2 × 5 mm beam, with the shorter dimension aligned along the pipe axis.

#### 2.1.3. High-heat-flux-exposed pipes   

Thermal break divertor mock-ups containing four tungsten tiles were manufactured and subsequently exposed to high-heat-flux conditions in the GLADIS test facility (Greuner *et al.*, 2007 ▸), while being actively cooled with pressurized water at 403 K (Lukenskas *et al.*, 2019 ▸; Greuner *et al.*, 2019 ▸). The heat flux in this facility is delivered by a hydrogen ion beam. Specifically, the following components were obtained: CCFE#7 (exposed to 25 MW m^−2^ + 100 cycles at 10 MW m^−2^, then 35 MW m^−2^ + 100 cycles at 25 MW m^−2^) and CCFE#8 (exposed to 25 MW m^−2^ + 100 cycles at 10 MW m^−2^). These components were sectioned longitudinally and, from one half, the tungsten was wire-cut away to retain only the CuCrZr pipe and a minimal amount of braze/interlayer. The thickness of each section was measured to account for it in the SANS data reduction.

In addition to these samples, a piece of pipe well away from the tungsten blocks, which had just been exposed to the brazing cycle, was measured at the same time. This sample is referred to as the ‘reference pipe’.

### 2.2. Small-angle neutron scattering   

SANS was performed at the ISIS Pulsed Neutron and Muon Source (Didcot, UK; https://www.isis.stfc.ac.uk/; Melnichenko, 2016 ▸).

Data were collected on the SANS2D instrument for reference coupons and the ZOOM instrument for MUPs and pipes in transmission geometry, *i.e.* with the neutron beam normal to the sample (Heenan *et al.*, 2011 ▸; ISIS Pulsed Neutron and Muon Source, 2021 ▸). In this geometry, the SANS data are generally insensitive to any surface layer contributions (*e.g.* oxide layers, surface roughness) extending less than ∼1000 Å. The beam footprint at the sample was collimated to 8 mm diameter on the reference coupons and 5 mm high by 2 mm wide on the MUPs and pipe sections, using cadmium masks located as close as possible to the samples. In the case of the MUPs, the long axis of the beam footprint was aligned across the diameter of the tube [see Fig. 2 ▸(*a*)], whereas in the case of the pipe sections it was aligned along the length of the tubes to allow measurements alongside either the top or bottom edge of the original component (by rotating the pipe sections ±45° into normal incidence) as well as through the centre [see Fig. 2 ▸(*c*)].

SANS2D and ZOOM are time-of-flight instruments, which means they utilize a range of neutron wavelengths (1.75–16.5 Å) to simultaneously probe a very broad range of *d* spacing (for the instrument configuration used, *d* is of the order of 8–3140 Å) and are thus ideal for the type of study performed here. However, examination of the transmission data revealed prominent Bragg edges at wavelengths shorter than 4.3 Å, a common occurrence in multi-phasic materials with crystalline regions, and so these wavelengths were excluded from the data reduction. In the figures below, the magnitude of the scattering vector *Q* = 2π/*d* = 4πsinθ/λ, where λ is the neutron wavelength and 2θ is the scattering angle. Scattering data were accumulated for 20–80 µA h (∼0.5–2 h) depending on the beam footprint and transmission data for 6–8 µA h (∼10 min).

For SANS to arise, there must be a contrast – a difference in scattering length density (SLD) – between sample components. The SLDs of Cr (3.01 × 10^−6^ Å^−2^) and Zr (3.07 × 10^−6^ Å^−2^) are so similar that the neutrons are unable to distinguish between Cr- and Zr-rich precipitates or phases. But it is likely that the dominant precipitate is a Cr-rich phase, especially given the low Zr content (Chen, Jiang, Jiang *et al.*, 2018 ▸; Abib *et al.*, 2019 ▸). Instead, here SANS arises from the contrast between Cr- and Zr-containing precipitates and a predominantly Cu matrix (SLD 6.55 × 10^−6^ Å^−2^). We note that the SLD of W is also 3.01 × 10^−6^ Å^−2^ and thus any residual scattering from the remains of the W monoblock would be indistinguishable from the Cr- or Zr-containing precipitates. Where the residual W can have an effect is through neutron absorption, as it has the largest absorption cross section of the four nuclei at 18.3 barns (Cr: 3.0 barns; Zr: 0.2 barns; Cu:3.8 barns). However, for comparison, the equivalent value for Cd – used in neutron shielding/masking – is 2520 barns. The measured SANS intensities, *I*(*Q*), were placed on an absolute scale by reference to the scattering from a standard sample.

Data reduction was performed using the Mantid framework (Mantid Project, 2013 ▸; Arnold *et al.*, 2014 ▸) in accordance with standard procedures for SANS2D and ZOOM. During processing, the data from the 2D detectors were radially averaged to one dimension. The curvature of the surfaces is not taken into account during the data reduction: as the neutron beam was symmetrically incident across the diameter of the MUP and the detector was 4 m behind the sample, it is a reasonable approximation to assume that the pipe surface is flat.

The reduced data were primarily fitted to a scattering function consisting of a combination of Porod’s law (Schmidt, 1991 ▸) and a single broad Lorentzian peak, as a function of the scattering vector magnitude *Q*:
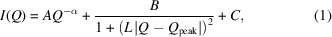
where α (the Porod exponent) is related to the degree of interfacial roughness, *L* (an Ornstein–Zernicke-like correlation length) is interpreted as analogous to the size of the precipitates, *Q*_peak_ (the peak position) is related to the separation between the centre of mass of the precipitates, *A* and *B* are scale factors, and *C* is the *Q*-independent background. The advantage of this function is that it does not presuppose a uniform shape for the precipitates (*e.g.* spheres) as others studying these alloys have done (Abib *et al.*, 2019 ▸), nor does it infer a mechanism for the phase separation (*i.e.* nucleation and growth versus spinodal decomposition). However, as morphological changes in the precipitates were evident from TEM work on these samples (Cackett *et al.*, 2018 ▸, 2021 ▸) and from some poor fits to equation (1), some of the same data were also fitted to scattering functions where the Lorentzian term in equation (1) was replaced by the form factors for homogeneous spheres or ellipsoids:
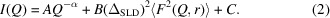
Here, the angle brackets denote an orientational average, *F*(*Q*, *r*) is the scattering amplitude and *r* is the form radius (*r* = *r*_sphere_ or *r*_ellipsoid_):
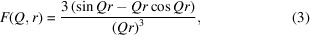


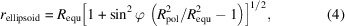

*R*_equ_ and *R*_pol_ are the equatorial and polar radii of the ellipsoid, respectively, and φ (over which the averaging is performed) is the angle between the rotational axis of the ellipsoid and **Q**. The ellipsoid is oblate if *R*_pol_ < *R*_equ_ and prolate if *R*_pol_ > *R*_equ_. In practice, an integration over a range of *r* was also performed (using a lognormal distribution) to allow for the effect of size dispersity. This model fitting was performed using the *SasView* software (version 5.0.4; https://www.sasview.org/). Further details of the models (‘broad_peak’, ‘sphere’ and ‘ellipsoid’) can be found in its comprehensive help documentation, either in-program or online. The average precipitate spacing in the ellipsoid model has been estimated as
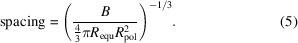
Equation (5) is valid when the scale factor *B* ≫ *A* in equation (2), under which condition *B* is approximately equivalent to the volume fraction of precipitates.

## 3. Results and discussion   

### 3.1. Reference coupons   

**Figure 3:**
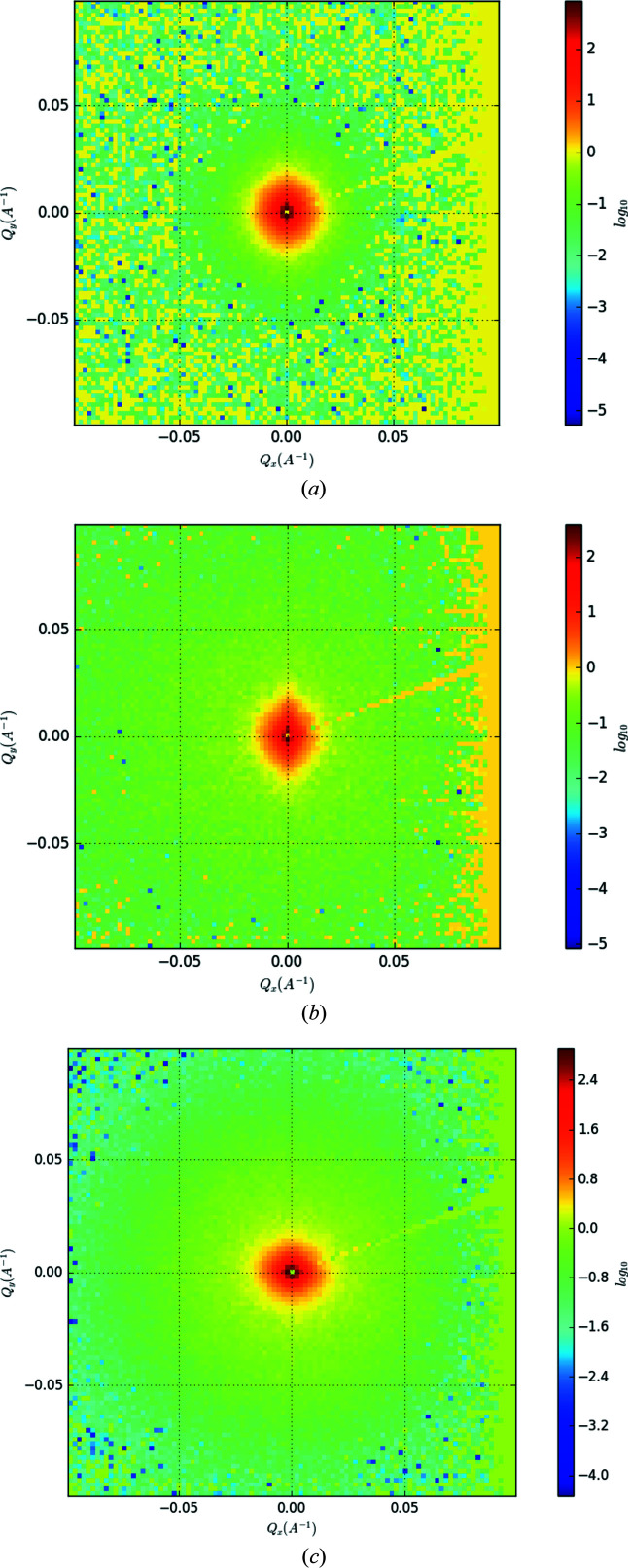
Two-dimensional scattering patterns for the (*a*) solution-annealed, (*b*) 753 K-aged and (*c*) 823 K-aged samples. Note the slight longitudinal anisotropy present in (*b*).

Fig. 3 ▸ shows the 2D scattering intensity against *Q* *_x_* and *Q* *_y_* for three different conditions, namely as received (solution annealed), 753 K and 823 K. A spherically symmetric pattern dominates the scattering – except for the optimally aged sample [Fig. 3 ▸(*b*)]. This scattering profile is slightly elongated in the *Q_y_* direction. Rotating the sample by 90° in the beam and remeasuring rotated the pattern as well, indicating that the precipitates in this CuCrZr coupon have some preferential alignment (along *Q* *_x_* in the figure). Since this anisotropy occurs only at the peak-aged condition, it might be related to the β′(II) precipitates, which are an intermediate between the initially formed β′(I) and the β precipitates characteristic of over-ageing as defined and observed by Chen, Jiang, Jiang *et al.* (2018 ▸).

**Figure 4:**
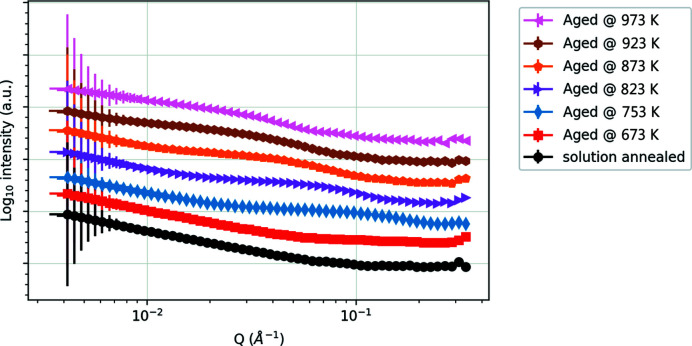
Radially integrated SANS data measured on SANS2D from the reference coupons for all heat-treatment conditions, showing the evolution of nanostructure in the alloy. The data are offset vertically for clarity.

For the 1D data reduction of this anisotropic profile, only the data within an azimuthal range extending ±30° either side of the meridian were used to capture the feature with adequate signal-to-noise ratio. An overview of the 1D intensity versus scattering data for all temperature conditions is shown in Fig. 4 ▸. There is a clear trend, consistent with the heat treatment of each sample. No distinct peak or characteristic features are present in the solution-annealed sample. After the 673 K treatment, a peak starts to emerge, becoming more pronounced after the 753 K treatment. Ageing at higher temperatures shifts this feature to lower *Q* values (longer length scales). After ageing at 923–973 K, a second peak emerges at higher *Q* (∼0.1 Å^−1^) than the first one (∼0.03 Å^−1^).

**Figure 5:**
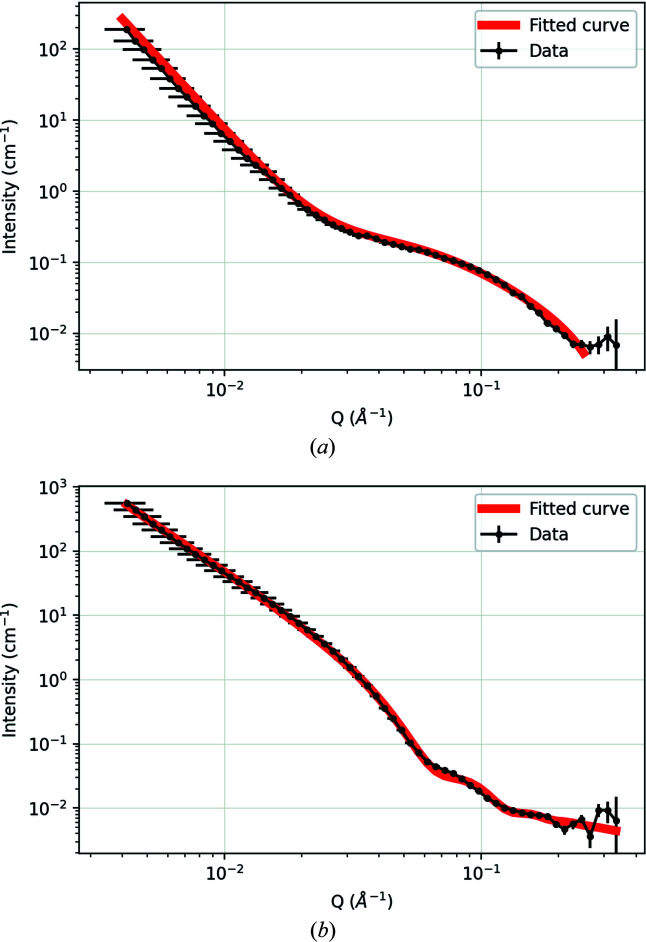
Examples of model fitting of the SANS data from the reference coupons: (*a*) 753 K-annealed sample fitted to equation (1); (*b*) 973 K-annealed sample best obtained fit to equations (2)–(4) [Disp-formula fd4].

Fitting these 1D spectra with equation (1) where possible [see Fig. 5 ▸(*a*)] shows a variation of the precipitate interface, as characterized by the Porod exponent (Table 1 ▸). An α value of 4 indicates a perfectly smooth interface, whereas a value of 3 indicates a much rougher interface (Schmidt, 1991 ▸; Teixeira, 1988 ▸). Initially the precipitates are coherent with the matrix (Porod exponent close to ideal, *i.e.* 4), but they become incoherent with over-ageing and thus tend towards 3. This has been independently observed by TEM on peak-aged and over-aged samples (Batra *et al.*, 2003 ▸; Chen, Jiang, Jiang *et al.*, 2018 ▸; Chen, Jiang, Liu *et al.*, 2018 ▸; Jha *et al.*, 2021 ▸). Note that coherent and incoherent here refer to the condition of the precipitate interface, not the type of neutron–nucleus interaction.

Equation (1) produces a poor fit for the reference squares annealed at 923 and 973 K. These data are more cogently reproduced by the ellipsoid model in equations (2)–(4) [Disp-formula fd4] [Fig. 5 ▸(*b*)], where it can be seen that the apparent second peak in the SANS data at these higher temperatures is in fact a consequence of the oscillatory nature of the ellipsoidal scattering function (here smeared by size polydispersity). This corresponds to the findings from TEM and APT data, which indicate aligned elongated precipitates upon over-ageing (Chbihi *et al.*, 2012 ▸; Chen, Jiang, Jiang *et al.*, 2018 ▸; Cackett, 2020 ▸; Jha *et al.*, 2021 ▸). The SANS data therefore seem to support a gradual morphological evolution from spherical precipitates at or below peak ageing, to oblate ellipsoidal precipitates (axial ratio ∼1:2) at 873 K, to more rod-like precipitates (axial ratio ∼1:12) at 973 K. All of our model fitting indicates quite broad precipitate size distributions (*e.g.* 25–60% of the median radius).

Table 1 ▸ shows a comparison of the fitting parameters extracted from SANS with data from TEM on sister samples that have undergone exactly the same heat treatment (Cackett *et al.*, 2018 ▸), as well as other TEM and APT data from the literature (Chbihi *et al.*, 2012 ▸; Chen, Jiang, Jiang *et al.*, 2018 ▸). The precipitate sizes are well correlated between SANS and TEM/APT, at least for the under-aged or peak-aged samples; only SANS is able to accurately resolve the highly anisotropic precipitates. The data also correspond well to those determined from other SANS studies on a CuCrZr alloy of similar composition, which extracted a spherical diameter of 3.2 nm in samples annealed at 823 K for 4 h (Abib *et al.*, 2019 ▸). The separation distance (related to the number density of precipitates) shows a better correspondence at higher temperatures, although for the case of TEM these are often an estimate based on the specimen thicknesses in a particular sample region, whereas SANS provides a millimetre-scale overview through the sample thickness. As the temperature and amount of over-ageing increases, especially on the 923 and 973 K samples, the quality of the fit with a single Lorentzian peak deteriorates, suggesting that this model is no longer appropriate. This could be due to multiple types of scatterers/precipitates and/or their shape becoming more pronounced, as observed elsewhere (Chbihi *et al.*, 2012 ▸; Jha *et al.*, 2021 ▸) and discussed above.

These experiments on carefully controlled samples demonstrate that SANS can clearly distinguish between different CuCrZr heat treatments, with an interpretation of the results comparable to TEM and APT. Similar conclusions were drawn for FeCr phase separation data (Xu *et al.*, 2016 ▸).

### 3.2. Manufacturing mock-ups   

**Figure 6:**
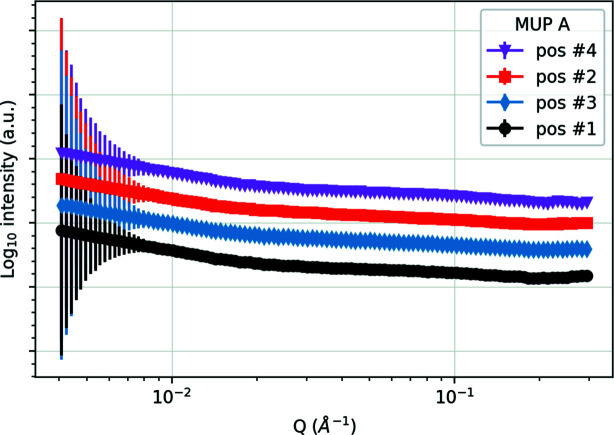
Radially integrated SANS data measured on ZOOM at the four positions on MUP A [see Fig. 2 ▸(*a*) for locations]. The data are offset vertically for clarity.

**Figure 7:**
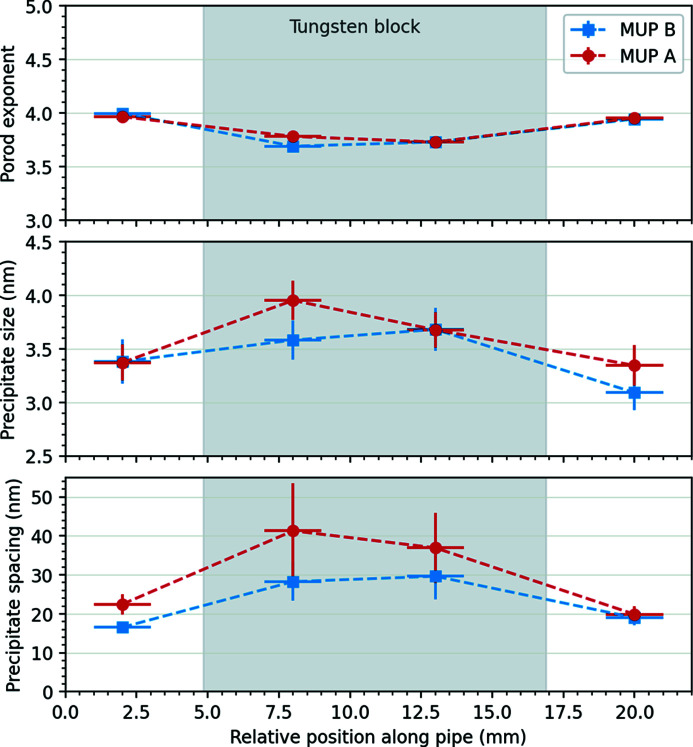
Parameters extracted from fitting SANS data from the four measurement areas on MUP A and MUP B to equation (1): Porod exponent (top); precipitate size (middle); precipitate spacing (bottom). The horizontal error bars reflect the width of the beam. The vertical error bars represent one standard deviation on the parameters as derived from the model fit. The position of the tungsten block is marked by a grey rectangle.

Fig. 6 ▸ shows the SANS data for the four different measurement points on MUP A, which should be comparable to the 753 K coupon sample. The derived parameters of fitting equation (1) to the data from MUP A and MUP B are shown graphically in Fig. 7 ▸.

The values for the Porod exponent and precipitate diameter on the copper pipe on either side of the tungsten block are broadly in line with the values for the peak-aged reference samples in Section 3.1 and Table 1 ▸, the precipitate spacing being slightly smaller. There appears to be a slight variation in all three parameters under the tungsten block, consistent with the trend on over-ageing from Section 3.2. The variation in precipitate size [Fig. 7 ▸ (bottom)] is around one or two times the lattice constant of crystalline copper, so it is significant. This would be consistent with a subtly different thermal response of the CuCrZr due to the locally different thermal mass, resulting in a slower cooling rate after the solution annealing, which promotes the nucleation of secondary phases (Park *et al.*, 2008 ▸).

These experiments confirm that the brazing cycle heat treatment from Fig. 1 ▸(*b*) does indeed produce near-optimum properties in the CuCrZr, with limited difference between whether the pipe goes through one or two braze heat treatment cycles. There is, however, a slight over-ageing present under the tungsten blocks.

### 3.3. High-heat-flux-exposed pipes   

**Figure 8:**
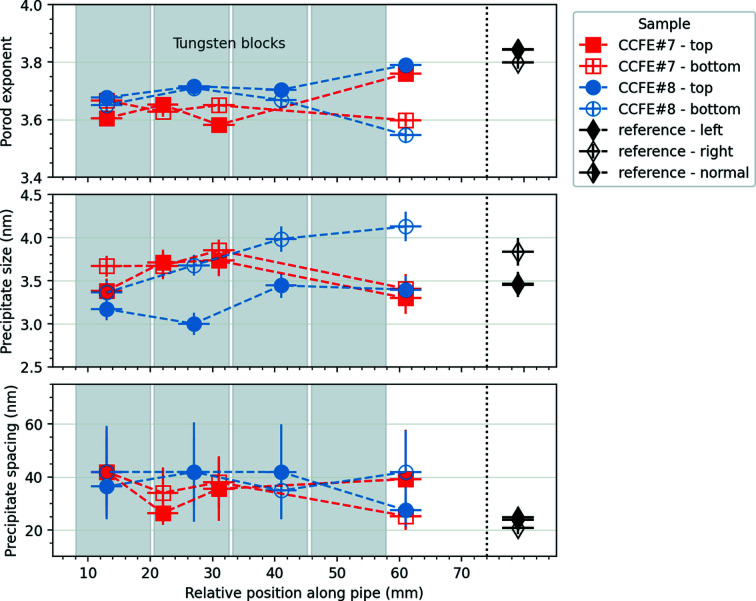
Parameters extracted from fitting the SANS data from the high-heat-flux-exposed samples and reference pipe: Porod exponent (top); precipitate size (middle); precipitate spacing (bottom). The horizontal error bars reflect the width of the beam. The vertical error bars represent one standard deviation on the parameters as derived from the model fit. The reference data in all three orientations are plotted on the same *x*-axis scale for comparison, although in some cases values overlap. Filled symbols refer to the heat-flux-exposed top surface; open symbols refer to the bottom of the MUP pipe samples. The positions of the tungsten blocks are marked by grey rectangles.

The results of fitting the SANS data for the reference pipe and high-heat-flux-exposed pipes are shown in Fig. 8 ▸. In the case of the reference pipe, all physical parameters should be the same regardless of the pipe orientation (*i.e.* left edge, right edge or centre), so the uncertainties on the fitted values give an indication of the accuracy to the underlying property. For the Porod exponent and precipitate spacing the values are close together, whereas there is a ∼0.25 nm difference on the fitted precipitate diameter between the right-hand side and the left and centre values. This is just below the difference observed in Section 3.2 under the tungsten block and around the value of the lattice constant of copper, so it could be a real local deviation.

For the high-heat-flux-exposed samples, there does not appear to be a distinct trend between the top or front (exposed to the high heat flux) and the bottom or back (not facing the particle beam). This is in line with the expectation from finite element analysis modelling, showing that the temperature at the top of the pipe remains below 633 K even when loaded to 20 MW m^−2^, while the bottom of the pipe remains at the coolant temperature of 403 K (Domptail *et al.*, 2020 ▸). At this temperature, no changes to the precipitates are anticipated (Kalinin *et al.*, 2007 ▸). Only the Porod exponent appears to be different between top and bottom away from the tungsten blocks, with the exponent from the heat-exposed side corresponding well to the values found in coupons aged at a similar temperature and the reference pipe.

These data, combined with the reference pipe data, imply that there is no meaningful temperature difference between the front and back of the MUPs during high-heat-flux exposure in the GLADIS facility and that the mechanical properties in the as-fabricated state remain more or less intact under these operating conditions. This demonstrates that the thermal break concept and its manufacturing route produce and retain the optimum CuCrZr structure.

## 4. Conclusion   

In summary, we have demonstrated that SANS can be used to resolve the state of CuCrZr precipitates in fusion reactor divertor components at a gauge volume of millimetre scale, with minimal sample preparation required. On reference samples with different heat treatments, there is good correspondence to APT and TEM data, except for highly anisotropic precipitates, which only SANS can accurately resolve.

The manufacturing method for the thermal break EU divertor concept studied here, namely brazing the solution-annealed CuCrZr pipe, followed by a gas quench and an ageing treatment, is demonstrated to give near-optimal microstructural properties, with variations underneath the tungsten monoblock, as detected by SANS, suggesting slight over-ageing. The near doubling of the precipitate spacing would alter the strength offered by the precipitates, though the error bars on these numbers are large and it is difficult to draw definitive conclusions.

Importantly, there was no significant variation or deterioration of the microstructure observed in high-heat-flux-exposed samples, confirming that the mechanical properties of as-manufactured divertors can be assumed to remain valid under standard operating conditions.

The sensitivity of SANS for precipitate coherency with the matrix shows promise for investigating neutron-irradiated samples over millimetre scales. This will aid in elucidating the irradiation-induced hardening and softening behaviour, which has been difficult to understand with TEM or APT.

## Supplementary Material

SANS data reduced, radially-averaged to 1D and normalized for effective sample thickness for CuCrZr coupons, manufacturing mock-ups and test mock-ups: https://doi.org/10.6084/m9.figshare.14587818.v1

## Figures and Tables

**Table 1:** Comparison of SANS data with TEM and APT data on precipitate size and spacing The average spacing was derived from the APT or TEM precipitate density *n* as *n* ^−1/3^. The SANS fitting model is denoted by L = Lorentzian, S = sphere and E = ellipsoid. Note that the fit of the Lorentzian model is particularly poor for the 873, 923 and 973 K data, so these values are omitted. The ellipsoid model for the 973 K data does not allow the calculation of the precipitate spacing, because the condition *B* ≫ *A* in equation (2) is not valid.

Material condition	SANS fitting model	SANS Porod exponent	SANS precipitate size (nm)	SANS precipitate spacing (nm)	TEM/APT precipitate diameter (nm)	TEM/APT precipitate spacing (nm)	Reference
As received	L	3.83 ± 0.01	N/A	N/A	N/A	N/A	SANS: this work; TEM: Cackett *et al.* (2018 ▸, 2021 ▸); Cackett (2020 ▸)
2 h aged 673 K	L	3.89 ± 0.01	1.11 ± 0.11	N/A	1.56	N/A	
2 h aged 753 K	L	3.91 ± 0.02	3.27 ± 0.13	35.81 ± 3.06	3.18	13.3	
	S	3.67 ± 0.01	2.40 ± 0.22				
2 h aged 823 K	L	3.84 ± 0.94	10.31 ± 0.05	20.26 ± 0.08	7.04	20.7	
2 h aged 873 K	S	4.18 ± 0.01	7.70 ± 0.22	–	12.02	37.9	
	E	4.18 ± 0.01	(6.92 ± 0.03) × (14.28 ± 0.07)	84.0	–	–	
2 h aged 923 K	E	3.22 ± 0.02	(10.53 ± 0.08) × (23.05 ± 0.53)	144.8	15.99	60.0	
2 h aged 973 K	E	2.63 ± 0.01	(0.87 ± 3.59) × (10.08 ± 0.06)	–	33.17	104.4	
MUP A (avg)	L	3.85 ± 0.12	3.59 ± 0.29	30.14 ± 10.57	–	–	
MUP B (avg)	L	3.84 ± 0.15	3.43 ± 0.26	23.36 ± 6.56	–	–	
Pipe reference (avg)	L	3.83 ± 0.03	3.58 ± 0.22	23.11 ± 2.14	–	–	
CCFE#7 top (avg)	L	3.68 ± 0.09	3.50 ± 0.20	34.10 ± 6.92	–	–	
CCFE#7 bottom (avg)	L	3.64 ± 0.03	3.69 ± 0.18	34.79 ± 7.12	–	–	
CCFE#8 top (avg)	L	3.72 ± 0.05	3.25 ± 0.21	36.90 ± 6.82	–	–	
CCFE#8 bottom (avg)	L	3.64 ± 0.07	3.79 ± 0.34	40.13 ± 3.51	–	–	

5 h aged 713 K		–	–	–	–	9.52 ± 0.25	Chbihi *et al.* (2012 ▸)
10 h aged 713 K		–	–	–	–	15.47 ± 0.77	

2 hr aged 723 K		–	–	–	4	–	Chen, Jiang, Jiang *et al.* (2018 ▸)

Prime aged (PA)		–	–	–	–	15.7	Edwards *et al.* (2007 ▸)
PA + 873 K/2 h		–	–	–	–	38.2	
PA + 973 K/4 h		–	–	–	–	87.4	

3 h aged 733 K		–	–	–	2.2–2.8	15.7–17.1	Hatakeyama *et al.* (2008 ▸)

Reference		∼4	2.2 ± 0.4	–	–	–	Abib *et al.* (2019 ▸)
ECAP + 4 h/823 K		∼4	3.2 ± 1.0	11.6	2.5	7.09	
